# Hallucinations: Etiology and clinical implications

**DOI:** 10.4103/0972-6748.62273

**Published:** 2009

**Authors:** Santosh Kumar, Subhash Soren, Suprakash Chaudhury

**Affiliations:** Department of Psychiatry, Ranchi Institute of Neuropsychiatry and Allied Sciences, Kanke, Ranchi, Jharkhand, India

**Keywords:** Etiology, Hallucinations, Psychopathology

## Abstract

The literature on hallucinations is reviewed, including history; theoretical background from physiological, biochemical and psychological points of view; classification; causation; presentation in different psychiatric and neurological disorders and in normal persons. The available evidence suggests that hallucinations result from a failure of the metacognitive skills involved in discriminating between self-generated and external sources of information. Management of hallucinations is briefly discussed.

Hallucinations are intriguing psychological phenomena that have a number of important clinical, theoretical and empirical implications; they are also among the most severe and puzzling forms of psychopathology. Usually regarded as characteristic of psychoses, they are found in a wide range of medical and psychiatric conditions. Moreover, a substantial minority of normal individuals report hallucinatory experiences. Phenomenologically they are the commonest and the most important disorder of perception. Although the exact cause and pathogenesis of hallucinations are not known, evidence points toward multiple etiological factors for the hallucinatory phenomenon.

## PSYCHODYANMIC APPROACH

Freud (1953) felt that hallucinations are very similar to dreams and that both conditions represent a psychotic state in which there is a complete lack of time sense. In this process, thoughts are transformed into visual images, mainly of a visual sort, that is, word presentations are taken back to corresponding “thing” presentations. According to Kolb and Brodie (1982), hallucinations represent a breakthrough of preconscious or unconscious material into consciousness in response to certain psychological situations and needs, e.g., wish fulfillment, enhancement of self-esteem, guilt feelings. The contents of hallucinations are thought to reflect their psychodynamic significance. Hallucinations often occurring with delusions during psychotic states may represent the concrete symbolic expression of delusional ideas that are seeking other routes of expression.

## PSYCHOPHYSIOLOGIC APPROACH

### Neurophysiologic hypothesis

Hughlings Jackson (1932) suggested that hallucinations occur when the usual inhibitory influences of the uppermost level are impeded, thus leading to release of middle-level activity, which takes the form of hallucinations. This model is known as disinhibition model. Penfield *et al*. (1950) demonstrated that electrical stimulation of certain cortical or subcortical structures induced different types of hallucinations. He proposed the theory of abnormal brain excitation as a mechanism of production of hallucinations. The neurophysiologic dissociation theory (Marrazzi, 1970) proposes that hallucinations result from a dissociation between primary sensory cortex and cortical association areas which exert a regulatory influence on the former. The perceptual release theory (West, 1975) postulated the presence of a censorship mechanism in the brain which actively excluded from the consciousness the majority of sensory information that is received continually by the brain. But the censorship mechanism can operate only when there is constant flow of sensory inputs. If by any chance there is stoppage or impairment of sensory input (e.g., in case of excessive affects during “functional psychosis,” prolonged periods of sensory deprivations), then earlier perception or memory traces emerge into the conscious, and the individual experiences hallucinations. This explains the occurrence of hallucinations following specific sensory modality deprivation.

### Neurotransmitter hypothesis

#### Dopamine

In schizophrenia (SCZ), there is evidence that very high levels of dopamine in the limbic system play a major role in emergence of hallucinations and delusions. Antipsychotic medications, which block central dopamine activity, alleviate the hallucinations of psychosis. Drugs with strong dopaminergic effect, such as L-dopa, methylphenidate, bromocriptine, pramipexole and piribedil, may induce hallucinations. D-amphetamine, a direct dopamine agonist, may also induce psychosis and hallucinations. The fact that hallucinations were also described in Parkinson’s disease before the introduction of L-dopa indicates that not only hyperdopaminergic states but also hypodopaminergic states, presumably due to progressive loss of dopamine projections to the cortex, can induce hallucinations.

#### Acetylcholine

A deranged cholinergic neurotransmission has also been involved in the pathophysiology of hallucinations. For example, alteration of consciousness and hallucinations have been described widely since ancient times for members of the Solanaceae family of plants (belladonna and dhatura), which contain scopolamine, atropine and other antimuscarinic agents. Hallucinations occur in about 30% of patients with Alzheimer’s disease and 60% of patients with Lewy body dementia, which are characterized by reduction in acetylcholine and abnormalities in nicotinic and muscarinic receptor expression.

#### Serotonin

Serotonin has also been implicated in the causation of hallucinations, based on the fact that a number of hallucinogenic drugs, like lysergic acid diethylamide (LSD), mescaline, psilocybin and ecstasy, appear to act, at least in part, as serotonin 5 HT_2A_ receptor agonist or partial agonists. In addition, hallucinations have been reported as side effects of Selective serotonin reuptake inhibitors (SSRIs), which increase the availability of serotonin in the synaptic cleft.

#### Glutamate

A possible role of glutamate in hallucinations is suggested by the finding that glutamate antagonists like phencyclidine and ketamine can induce hallucinations. This has led to the hypothesis that psychotic symptoms may in part be attributed to hypofunction of NMDA receptors.

#### GABA-A

PET and SPECT studies using GABA-A receptor ligands showed that the intensity of hallucinations was strongly associated with diminished GABA-A binding, specifically in the left medial temporal region.

#### Electroencephalographic studies

Using cognitive tasks involving speech production and perception, Heinks-Maldonado *et al*. (2007) suggested that in healthy subjects, EEG synchrony preceding speech reflects the action of a forward model system, which dampens auditory responsiveness to self-generated speech. However, the dampening was deficient in SCZ patients with hallucinations. Hubl *et al*. (2007) investigated the responsiveness of the auditory cortex during hallucinations in SCZ patients using the N100 evoked potential in response to auditory stimulation. During hallucinations, the N100 amplitude was smaller, presumably because of a reduced left temporal responsivity. They concluded that the findings indicate competition between auditory stimuli and hallucinations for physiological resources in the primary auditory cortex. Abnormal activation of the primary auditory cortex may thus be a constituent of auditory hallucinations.

### Neuro-imaging studies

#### Structural imaging

In a systematic review of 46 studies (including 1444 patients with SCZ and 1327 controls) on superior temporal gyrus (STG), volumetry MRI studies in SCZ revealed that left STG or subregions appear to be more involved in the generation of hallucinations and thought disorder than right sides. The majority of 5 follow-up studies found evidence of progressive changes in volumes (Sun *et al*., 2009). Volume reductions in the prefrontal and cerebellar cortices have also been reported and may be associated with impairment in the monitoring or awareness and volition of internal speech. MRI brain scans of 66 patients of type I bipolar disorder and 66 controls indicated that hallucinations and positive symptoms were associated with gray matter reduction in the left middle temporal gyrus (Stanfield *et al*., 2009).

#### Functional imaging

Stephane *et al*. (2000) found increased rCBF in the left temporal region in hallucinating patients. McGuire *et al*. (1993) reported that hallucinatory state was associated with greater blood flow in language-related areas, significantly so in a region corresponding to Broca’s area and anterior cingulate and left temporal cortices. Nonpsychotic visual hallucinations in Parkinson’s disease (PD) were associated with hypoperfusion in the right fusiform gyrus and hyperperfusion in the right superior and middle temporal gyri. These temporal regions are important for visual object recognition, and these rCBF changes are associated with inappropriate visual processing and are responsible for nonpsychotic visual hallucinations in PD (Oishi *et al*., 2005). Using PET during auditory verbal hallucinations (AVH), patients demonstrated a significant activation of the supplementary motor area, anterior cingulum, medial superior frontal area and cerebellum. Activation was also observed in the left superior frontal area, right superior temporal pole and right orbitofrontal region (Parellada *et al*., 2008). Stephane *et al*. (2006) suggest that the abnormal laterality of the supplementary motor area activity accounts for the failure to attribute speech generated by one’s own brain to one’s self and that the activation of Wernicke’s area accounts for the perceptual nature (hearing) of the patient’s experience. Lahti *et al*. (2006) using PET reported a positive correlation between hallucinations and activation of anterior cortex and a negative correlation with hippocampus-parahippocampus. Functional MRI revealed that AVHs were associated with activation in the right STG and posterior insula, the right middle temporal gyrus, and hippocampus/parahippocampal gyrus. Somatic hallucinations were associated with activation in the thalamus, insula and posterior cingulate gyrus bilaterally. Further activation was evident in the left middle frontal gyrus, the precentral gyrus, twin foci in the postcentral gyrus, inferior parietal lobule, the middle and superior temporal gyri with right-sided inferior frontal gyrus, and precuneus (Hoffman *et al*., 2008; Ford *et al*., 2009). Functional imaging studies have consistently shown the involvement of associative sensory areas. Moreover, the hallucinating brain may be characterized by stronger inputs from subcortical centers (especially the thalamus); reduced control by the dorsolateral prefrontal cortex; and aberrant activation of dorsal anterior cingulate, which is involved in source monitoring.

#### Anatomical correlates

Hallucinations cause sensory modality-specific activation in cerebral areas involved in normal sensation. A disturbance in perception of speech seems to have a central role in occurrence of auditory hallucinations. Anatomically, auditory hallucinations appear to involve primary and association cortices; Broca’s and Wernicke’s areas; subcortical, paralimbic, limbic regions; ventral striatum; and thalamus. Furthermore, they are suggested to be associated with the dysmodulation of the information flow from ventral striatum to thalamus and cortex caused by increased dopaminergic activity in mesolimbic pathway. Hugdahl *et al*. (2008) proposed that auditory hallucinations are internally generated speech perceptions that are localized in the left temporal lobe, in the left peri-Sylvian region. If hallucinations are misidentified perceptions originating in the speech perception area of the left temporal lobe, then hallucinating patients should have problems identifying a simultaneously presented external speech sound, especially when the sound is presented lateralized to the left hemisphere. Using a dichotic listening paradigm with pair-wise presentations of consonant-vowel syllables, they showed that patients with schizophrenia who experience frequent and/or intense auditory hallucinations fail to show an expected right ear advantage (REA), which may be indicative of a functional deficit in this brain region. Patients who experience frequent hallucinations are impaired in their ability to use top-down cognitive strategies to overcome a ‘bottom-up stimulus’-driven right ear effect when instructed to focus attention on the left or right ear stimulus. The behavioral data are supported by MR brain imaging data looking for pathology of gray matter density in patients with schizophrenia, particularly in the left hemisphere. The reviewed studies revealed significant reductions in gray matter density in the left peri-Sylvian region, including thinning of the cortical mantle, also supported by altered neuronal activation as seen in the fMRI results.

#### Cognitive perceptual theories

Hallucinations can be seen as erroneous perception or “sensory deception.” Hence researchers have been investigating the integrity of perceptual functions in persons with hallucinations. Basically, 2 approaches can be distinguished. The first is concerned with bottom-up, or data-driven, perceptual processing, such as deficits in the processing of incoming visual stimuli in primary visual cortex; hearing impairment and auditory hallucinations associated with sensory impairment in older people; or the emergence of hallucinations in sensory deprivation. The second approach is concerned with top-down, or conceptual, processing in perception. This refers to processes that contribute to perception that do not originate in the external world but in the brain of the perceiver. Examples of such top-down factors are prior knowledge, perceptual expectations, attentional modulation and mental imagery. Bentall (1990) proposed that discrimination between internal and external sources of information is a metacognitive skill called source monitoring. Hallucinations result from failure of this skill. Hoffman *et al*. (1994) using psycholinguistic approach postulated that auditory hallucinations stem from disruption in language production caused by parasitic memory. David (1994) linked verbal hallucinations to inner speech. He argued that inner speech is produced and controlled by an inner-voice-inner-ear system, and the disruption of the system may lead to verbal hallucinations. Hamsley postulated that normal perception is dependant on an interaction between current sensory input and regularities that have been detected in previous sensory input. Memory of previous regularities and the current context result in ‘expectancies’ or ‘response biases’ towards the current sensory input. The central defect in hallucinations is the reduction of the influence of regularities of past experiences on current perception. This weakened influence would result in ambiguous and unstructured sensory input. Thus the unstructured sensory input would fail to inhibit the emergence of material from long-term memory into awareness; its emergence would be experienced as hallucinations (David, 1994).

#### Perception and attention deficit model

Collerton *et al*. (2005) proposed a PAD model of complex visual hallucination, in which the hallucinatory images generally occur in the focus of the visual field and are seen against the background of the existing visual scene. According to the PAD model, both sensory impairment and attentional abnormalities are needed for hallucinations to arise. More specifically, a combination of impaired attentional binding and poor sensory activation of a correct proto-object, in conjunction with a relatively intact scene representation, biases perception to allow the intrusion of a hallucinatory proto-object into a scene perception. Proto-objects refer to holistic or part-based abstracted object representations that are segmented from visual information and act as candidates for further processing but are at such an early processing stage that they have not yet entered conscious awareness. Such proto-objects are in multiple competitions for further processing. The interplay of top-down and bottom-up biasing information will eventually determine which proto-object will “win” and enter conscious awareness. Cholinergic dysfunction may result in a failure to properly integrate sensory information (bottom-up) and prior expectation (top-down). The PAD model proposes that impaired attentional binding is due to abnormal lateral frontal activity; poor sensory activation of a correct proto-object is due to abnormal ventral visual stream activity; and intrusion of a hallucinatory proto-object is mediated by increased temporal versus frontal activity. Diederich *et al*. (2005) suggested that visual hallucinations should be considered a dysregulation of the gating and filtering of external perception and internal image production. Contributive factors for their model include poor primary vision, reduced activation of primary visual cortex, aberrant activation of associative visual and frontal cortex, lack of suppression or spontaneous emergence of internally generated imagery through the ponto-geniculo-occipital system, intrusion of rapid eye movement dreaming imagery into wakefulness, erratic changes of the brainstem filtering capacities through fluctuating vigilance and medication-related overactivation of mesolimbic system. Not all of these have to be present, and different combinations will lead to differences in phenomenology. Llinas and Pare (1991) suggested that conscious perception is subserved by intrinsic activity in thalamocortical circuits that is constrained or modulated by sensory input. They considered the primary difference between conscious perception and dream imagery to be found in the weight given to sensory input, which is a large weight in conscious perception, and hence sensory factors largely determine the final percept, whereas this weight is negligible in dream imagery. Integrating the sensory impairment approach with the top-down approach, Behrendt and Young (2004) viewed hallucinations as “underconstrained perceptions” that arise when the impact of sensory input on thalamocortical circuits is reduced. If sensory constraints (i.e., bottom-up processing of incoming information) are weak, e.g., due to sensory impairment, attentional mechanisms (i.e. top-down influences) may become the dominant modulatory influence on thala mocortical oscillatory activity that gives rise to conscious percepts — in this case, hallucinations.

#### Neural network model

To test different hypotheses regarding neurocognitive basis of hallucination, Hoffman and McGlasher (2006) developed a neural network simulation. Their model specifically concerns auditory hallucinations as typically reported in people with schizophrenia and is based on 2 popular hypotheses regarding neurobiological basis of schizophrenia: (a) overzealous pruning of synapses that are an extension of normal developmental pruning during adolescence and (b) alterations in the dopaminergic neuromodulatory system in schizophrenia. To assess and compare these 2 hypotheses, a computer simulation of some aspects of speech perception was developed. This system was found to produce spontaneous percepts simulating hallucinated speech when the working memory component was excessively pruned or when neuronal responses were modulated to simulate a hyperdopaminergic system. Comparing the performance of the network with that of actual hallucinating patients and normal controls while tracking (repeating while simultaneously listening to) speech that was phonetically degraded, revealed that the neural network simulation producing the best match to speech-tracking performance of human hallucinators was an overpruned system with compensatory hypodopaminergic adjustments.

#### Metacognitive process: Reality monitoring and metacognitive beliefs

Metacognition refers to “thinking about thinking”or to beliefs and attitudes held about cognition. Most recent models of hallucinations assume that hallucinations are related to misattribution of private events. These private events may include a number of types and modalities, such as inner images, inner speech, voices, intrusive thoughts, vivid daydreams and bodily sensations. Different approaches have attempted to explain this misattribution, with the most prominent of these relating hallucinations to a misattribution of inner speech, problems in source monitoring, and relations between hallucinations and beliefs. Recent cognitive models of hallucination have attributed these misattributions to cognitive bias or cognitive deficit. Cognitive biases are present when some forms of information are processed preferentially in comparison to others. Examples of cognitive bias include the preferential recall of negative information in patients with depression. Cognitive deficits occur when there are disruptions of specific cognitive functions, including, for example, problems with working memory, inhibition or attention. It is probable that disturbances in cognitive processes as a result of cognitive deficits (i.e., bottom-up factors) and cognitive bias factors (top-down factors) are both responsible for hallucinations.

#### Reality monitoring and hallucinations – Bentall’s (1990) hallucination model

Some studies provide evidence for Bentall’s notion that hallucinators are impaired in their ability to discriminate between real and imagined events and reveal a specific bias towards attributing their thoughts to an external source. This externalizing bias seems related, at least in part, to the effects of both an inadequate use of cognitive effort cues and the emotional salience of stimuli (Laroi *et al*., 2004) on source-monitoring tasks.

#### Hallucinations and beliefs – Morrison’ hallucination model

Morrison is in agreement with the theory that hallucinations are internal cognitive events that are misattributed to an external source. His explanation for this misattribution is based on the fact that hallucinations are in some way linked to normal intrusive thoughts and that because of motivational factors these intrusive thoughts become externalized by the individual (Morrison *et al*., 1995). An important aspect of this theory concerns research showing similarities between intrusive thoughts and hallucinations, because the genesis of hallucinations is seen as a reaction to intrusive experiences. Like hallucinations, intrusions are also often experienced as ego dystonic and uncontrollable; share similarities in terms of form, content and triggers (e.g., stressful events); and are usually accompanied by subjective discomfort. The theory also states that the need to attribute intrusive thoughts to an external source is due to motivational factors. The presence of certain intrusive thoughts may lead to negative affect in the subject in the form of anxiety or cognitive dissonance. According to cognitive dissonance theory, dissonance occurs when 2 cognitions (e.g., thoughts, beliefs and feelings the person is aware of) contradict each other, resulting in an uncomfortable state from which an individual is motivated to escape. Morrison *et al*. (1995) argued that to reduce the levels of negative affect, the subject chooses to externalize the intrusive thought, resulting in hallucinations. Morrison also posited the important role that metacognitive beliefs may play in this misattribution process. Metacognitive beliefs are beliefs concerning one’s own mental process. This may include beliefs that mental events should be controllable, that thoughts are dangerous or harmful or that intrusive thoughts are acceptable and beneficial. When the occurrence of intrusive thoughts does not comply with the subject’s metacognitive beliefs, an aversive state of arousal results (cognitive dissonance), which the subject tries to escape by externalizing the intrusive thoughts (resulting in hallucinations), thus maintaining consistency in his belief system. A number of studies have found evidence for an association between metacognitive beliefs and the presence of hallucinations.

#### A comprehensive cognitive model of hallucinations

Alemon and Laroi (2008) presumed that an integrated network of cognitive processes may contribute to perceptual experience, and proposed that 4 different routes may result in a hallucination: (a) release phenomena due to lesions in sensory pathways/the arousal system (brainstem, thalamus); (b) irritative processes acting on cortical centers that integrate perceptual information; (c) unconstrained activation of the attentional spotlight; and (d) a cognitive route with key roles for affective state, top-down factors and source-/self-monitoring.

*Release phenomena*: Disturbed visual input may cause hallucinations by means of an abnormal release of central processing, though the level at which the release occurs is unclear. The implication of primary and sensory cortices seems straightforward, but changes have also been observed in subcortical brain areas (e.g., lateral geniculate nucleus) in patients with optic nerve lesions. The capacity for generating complex visual hallucinations may be located in the association cortex, which is released by restricted lesions, a loss of cortico-cortical inputs, and alteration of activity via the reticular activating system. In this model, release hallucinations would arise from damage to the component sensory input or its connecting pathway to sensory experience.

*Irritative processes*: Hallucinations caused by spontaneous activation of sensory cortical areas have been primarily described in epilepsy. The fact that focal cortical resection can result in complete remission of epileptic hallucinations indicates that these hallucinations cannot be attributed to a release phenomenon. According to this model, irritative processes would be confined to aberrant activation of the module sensory experience.

*Attentional spotligh*: The thalamic reticular nucleus (TRN) acts as a searchlight to guide attention to certain features of the environment and thereby intensifies processing of these features. The thalamus is acknowledged as one of the three important nodes in a network underlying selective attention that comprises the prefrontal cortex, parietal cortex and the thalamus. The TRN facilitates sensory processing by facilitating detection and discrimination through the initiation of burst-firing (when a population of neurons fires with a high-frequency burst in contrast to a continuous low-frequency baseline firing) in the lateral geniculate nucleus (LGN). Although the encoding of an externally presented stimulus becomes distorted during burst-firing, the background noise decreases significantly, thereby enhancing signal detectability. This model proposes that activation of the TRN in the absence of sensory input could explain the involuntary character of hallucinations. Such activation is possible via connections from the prefrontal cortex to the TRN, via feedback from sensory association cortex and via neurotransmitters such as dopamine and acetylcholine.

*Cognitive route*: The model assumes that hallucinations may arise from a cognitive route, in which emotion, top-down perceptual mechanisms and metacognitive functions (e.g., source- or self-monitoring) play a decisive role. The idea here is that an affective state (stress and anxiety) negatively influences monitoring systems, resulting in external misattribution of internally generated events, but can also influence top-down factors such as perceptual expectations. There is evidence that emotional factors can modulate verbal self-monitoring and source-monitoring. Neuro-imaging studies show that amygdala activation in response to emotional stimuli can boost activation of perceptual areas, which indicates that the affective state can influence perception and imagery.

**Figure 1:**
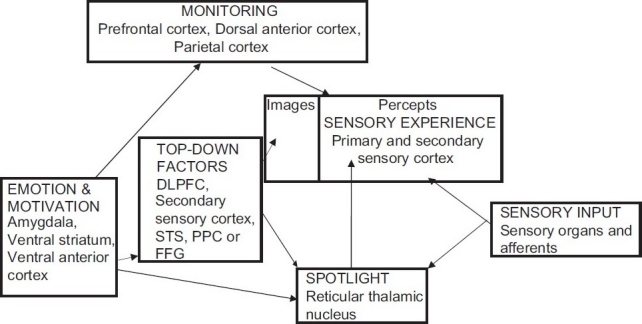
The role of emotion and motivation in cognitive processes. DLPFC - Dorsolateral prefrontal cortex; STS - Superior temporal sulcus; PPS - Posterior parietal sulcus; FFG - Fusiform gyrus

The top-down factors’ subsystem in the model refers to the influence of prior knowledge, perceptual expectations and attention on perception. Excessive activation of such top-down factors can “push” images to the right within the sensory experience box [Figure 1]. As a result, images will acquire stronger sensory and reality characteristics due to excessive top-down modulation and will therefore be more easily confused with bottom-up percepts. Activation of the thalamus in top-down attention has also been reported. Connections from the lateral prefrontal cortex to the thalamus may also mediate top-down influences in normal circumstances, in which bottom-up influences constrain the LGN activation by inputs to the reticular nucleus. Hyperactivation in the lateral prefrontal cortex or from emotion regulation areas in the orbitofrontal cortex to the reticular nucleus could lead to diminished control of the LGN and hence result in spontaneous burst activity, affecting projections to the sensory association cortex. These frontal regions target sensory tiers of the TRN to select relevant and motivationally significant signals. Alternatively, increased input into perceptual areas (e.g., Wernicke’s area) from frontal areas (e.g., Broca’s area) may affect the reticular nucleus via corticothalamic projections originating in the sensory association cortex. This mechanism of top-down influences largely overlaps with the attentional spotlight. In fact, it concerns a final common pathway (involving the TRN and the sensory association cortex) with different causative agents: Top-down factors such as expectations generated in prefrontal regions versus direct influences (e.g., through acetylcholine) in the third route (i.e., activation of the attentional spotlight) described above. It is also possible that top-down influences act without any intervention from the thalamus.

The monitoring subsystem in the model [Figure 1] refers to the metacognitive processes in which the cognitive system makes a decision regarding the source of perceptual events, that is, whether they are internally generated or externally presented. These 2 types of perceptual representations (images vs. bottom-up percepts) are shown in the sensory experience box [Figure 1]. The vertical line between images and percepts denotes the criterion for classifying a perceptual event as internally generated imagery versus externally presented sensory information. The gradient by which some experience is likely to be classified as an image or as a bottom-up percept is strongly affected by 2 factors: Vividness and sense of reality. Vividness refers to sensory, semantic and contextual details: A bottom-up percept is generally clear and rich in such details, whereas images are much fainter. The same holds for a sense of reality, which is of course influenced by vividness but is also influenced by expectations and metacognitive factors. Faulty monitoring of one’s mental processes could shift the criterion line to the left, thereby resulting in a misclassification of certain imagery experiences as being percepts originating from the outside. It is doubtful whether monitoring errors alone can cause hallucinations, as it is unclear how thought, inner speech or retrieved memories can be transformed into experiences with perceptual qualities just by virtue of their misattribution to an external origin. However, when they interact with overactive top-down systems, such biases could contribute to erroneous perceptual decision making. With regard to the neural basis of monitoring, the anterior cingulate cortex has a pivotal role. The premotor cortex and superior temporal gyrus (in the case of verbal material) are also involved in self-monitoring.

## CONCLUSION

Hallucination is a fundamental symptom in psychiatry. Two hundred years of research into this phenomenon has not yet answered the following questions: (1) Are hallucinations pathognomic of psychosis? (2) Can the presence of hallucinations as such or in different modalities and forms include or exclude certain diagnoses? (3) What is the neural substrate of hallucination? These questions are very basic to the understanding of mental diseases, and more research in both phenomenological and theoretical areas is necessary to fathom the secret. Conventionally, hallucinations are treated as psychotic features. However, there is ample evidence to support the fact that hallucination could be present in nonpsychotic conditions. Mechanism and nosological status of these conditions are yet not clear. Assessing cultural background in the evaluation of hallucination is important as the concept of reality varies across cultures and there is a possibility of culturally sanctioned hallucination. A part from effective pharmacological treatment, more awareness is needed regarding psychological treatment of hallucination, which can help us deal with refractory hallucinations.
